# Corneal Endothelial Cell Loss in Glaucoma and Glaucoma Surgery and the Utility of Management with Descemet Membrane Endothelial Keratoplasty (DMEK)

**DOI:** 10.1155/2022/1315299

**Published:** 2022-01-30

**Authors:** Neeru A Vallabh, Stephnie Kennedy, Riccardo Vinciguerra, Keri McLean, Hannah Levis, Davide Borroni, Vito Romano, Colin E Willoughby

**Affiliations:** ^1^Department of Corneal and External Eye Diseases, St Paul's Eye Unit, Royal Liverpool University Hospital, Liverpool, UK; ^2^Department of Eye and Vision Science, Institute of Life Course and Medical Sciences, University of Liverpool, Liverpool, UK; ^3^Department of Doctoral Studies, Riga Stradins University, Riga, Latvia; ^4^Genomic Medicine, Biomedical Sciences Research Institute, Ulster University, Coleraine, UK

## Abstract

The corneal endothelium has a crucial role in maintaining a clear and healthy cornea. Corneal endothelial cell loss occurs naturally with age; however, a diagnosis of glaucoma and surgical intervention for glaucoma can exacerbate a decline in cell number and impairment in morphology. In glaucoma, the mechanisms for this are not well understood and this accelerated cell loss can result in corneal decompensation. Given the high prevalence of glaucoma worldwide, this review aims to explore the abnormalities observed in the corneal endothelium in differing glaucoma phenotypes and glaucoma therapies (medical or surgical including with new generation microinvasive glaucoma surgeries). Descemet membrane endothelial keratoplasty (DMEK) is increasingly being used to manage corneal endothelial failure for glaucoma patients and we aim to review the recent literature evaluating the use of this technique in this clinical scenario.

## 1. Introduction

Glaucoma is a group of conditions with varying pathophysiological processes, which cause progressive optic neuropathy associated with characteristic structural damage to the optic nerve and associated visual field loss [1]. The condition can be caused by various pathophysiological processes. Worldwide, glaucoma is the leading cause of irreversible blindness worldwide with a global prevalence of 3.54% in people aged 40–80 years with the highest prevalence being in Africa [2].

Corneal endothelial abnormalities, including a reduction in cell count and morphology, have been detected in glaucoma patients [3]. The corneal endothelium is a monolayer of hexagonal cells, which plays a critical role in regulating corneal hydration and thus transparency [4]. The cells are highly interdigitated and possess apical junctional complexes that, together with abundant cytoplasmic organelles, including mitochondria, are indicative of their crucial role in active fluid transport [5]. The abnormal endothelial changes observed in glaucoma are due to multiple influences including the intraocular pressure (IOP), aqueous humour abnormalities, medication use, and surgical interventions [3]. This review article aims to describe the endothelial changes seen in glaucoma and the role Descemet membrane endothelial keratoplasty (DMEK) has in managing corneal endothelial cell loss in glaucoma patients.

In preparing this article, electronic database searches were performed for English publications using the following search terms; glaucoma (including different types of glaucoma), glaucoma surgery (including different types of glaucoma surgery), glaucoma medication (including different types of glaucoma topical therapy), corneal endothelium, and Descemet membrane endothelial keratoplasty (DMEK). The databases analysed included Medline, Embase, ClinicalTrials.gov, and PubMed. From the searches, all articles pertaining to the relevant topic were included in this review.

### 1.1. Assessment of the Corneal Endothelium

**Figure 1:**
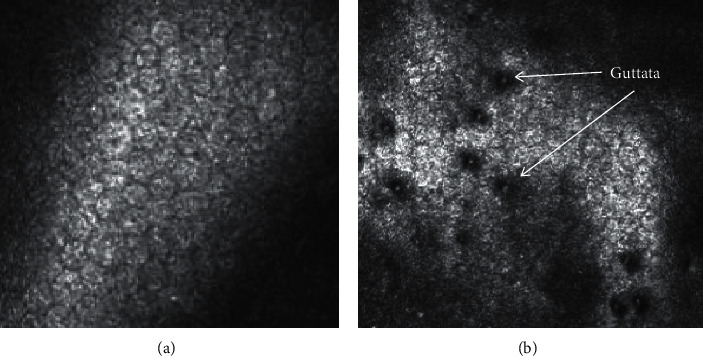
Confocal microscopy of corneal endothelial cells. (a) Normal endothelial cells with a regular hexagonal shape. (b) Fuchs endothelial corneal dystrophy shows a loss of defined hexagonal shape, increased cell size, and the formation of guttata (as labelled).

Slit-lamp biomicroscopy can detect macroscopic changes in the corneal endothelium and corneal endothelial diseases, such as Fuchs endothelial corneal dystrophy (FECD). Precise examination of corneal endothelial cell density (ECD) or cell count can be evaluated using, most commonly, specular microscopy or *in vivo* confocal microscopy (see Figure 1). Endothelial density is defined as the number of cells present in a 1 mm^2^ area.

### 1.2. Endothelium and Ageing

As mentioned, the corneal endothelium is a monolayer of hexagonal cells which maintain homeostasis of corneal hydration and transparency [4]. It sits upon a collagen basement membrane called Descemet's membrane. At birth, the Descemet's membrane is 3 µm thick, but this increases with age to an average of 13 µm at 70 years of age.

Corneal transparency is maintained by the active transport of ions across Na^+^/K^+^ ATPase pumps [6]. These pumps continually function to preserve the clarity of the cornea even if the IOP within the anterior chamber rises [7]. The integrity of the corneal endothelial monolayer is critical in maintaining this physiological function. The average corneal ECD during adulthood is 2500 cell/mm^2^, but natural ageing results in both the deterioration in number and morphology of these cells, including cell size and pleomorphism (loss of hexagonal shape) [8–10]. The rate of cell loss is constant throughout life at a rate of approximately 0.6% per year after the age of 18 [11]. This cell loss increases the permeability of the endothelial barrier and reduces its ability to pump fluid out of the corneal stroma and maintain corneal transparency [12]. Corneal endothelial cells show limited replicative ability *in vivo* [13].

Additionally, the ability of the Na^+^/K^+^ ATPase pumps deteriorates with age, decreasing from 32 µamps.cm^−2^ in people aged 60 years old to 22 µamps.cm^−2^ in those aged 90 (natural variation is ±6 µamps.cm^−2^) [14]. These age-related changes are well documented in the literature. Studies have reported that as the morphology of the corneal endothelial monolayer alters with age, it loses its barrier permeability as a result of a lower resistance at the intracellular junctions of the apical cell membranes [15]. Carlson et al. [16] reported in a study of corneas aged 5–79 years old that the number of hexagonal cells significantly decreased with age, but the number of pentagonal and heptagonal cells increased simultaneously [16]. In addition, they observed a 23% increase in endothelial permeability to fluorescein with age but found no differences in corneal thickness or pump rate. The flow rate of aqueous also remained stable. The authors concluded that as the cell morphology altered with age, the cell barrier became more permeable [16].

Age-related loss and changes of the corneal endothelium usually do not have much clinical relevance unless further cell loss is encountered in diseases such as FECD or surgical intervention. In these cases, the cell loss eventually overwhelms the ability for the corneal endothelium to maintain homeostasis leading to irreversible corneal oedema and blindness [12].

### 1.3. Influence of the Aqueous Humour on Corneal Endothelial Cells

The biological mechanisms responsible for the gradual loss of corneal endothelial cells are likely multifactorial including environmental, hormonal, and immune responses which may be responsible for cell migration, senescence, and apoptosis/necrosis of cells within the anterior segment during normal ageing [17]. As mentioned, corneal endothelial cells display limited proliferative capacity, although this is lower in older donors compared to younger ones [18]. A study on donor corneas also demonstrated that the length of the G1 phase of the cell cycle in corneal endothelial cells is longer in older donors (50 years) compared to younger donors (30 years) [13]. Transforming growth factor beta (TGF-*β*) may be partly responsible for this as it reportedly inhibits degradation of the G1-phase inhibitor, p27kip1, thus preventing the cells from entering into S-phase [19].

The anterior chamber and aqueous humour have immunosuppressive effects that permit inflammatory mediators and cells to circulate within the eye [20]. TGF-*β*2 [21] and *α*-melanocyte-stimulating hormone [22] are the dominant immunosuppressive molecules within the aqueous humour. Transforming growth factor (TGF)-*β*2 is known to be present within aqueous humour in normal eyes, which is in direct contact with the corneal endothelium [23]. Trivedi et al. demonstrated that significantly more TGF-*β*2 is present in the aqueous of older eyes without glaucoma [24].

Additional levels of inflammatory cytokines within the aqueous humour such as tumour necrosis factor (TNF), interleukin-1, and interferons (IFNs) are known to increase with age [25]. *In vitro,* they have been shown to induce apoptosis in corneal endothelial cells [25]. Intraocular surgery, such as cataract surgery, which is usually performed on older patients, has also been shown to increase cytokine levels associated with inflammation and apoptosis including interleukins, TNF-*α*, IFN-*γ*, TGF-*β*, and monocyte chemo-attractant protein-1 (MCP-1) [26, 27]. Cataract surgery can also lead to long-term alterations of the intraocular microenvironment in normal, glaucomatous [28], and FECD eyes [29].

## 2. Changes in the Corneal Endothelium Parameters in Glaucoma

Research has shown that TGF-*β* plays a crucial role in the aetiology of glaucoma, with significantly elevated levels identified in the anterior chamber of glaucomatous eyes [30]. TGF-*β* is a key mediator of fibrosis in all organs [31], through the excess production of extracellular matrix proteins including collagens and fibronectin [32, 33]. In addition, fibroblasts transform into highly contractile myofibroblasts, as demonstrated by the expression of alpha smooth muscle actin (*α*-SMA) [34–36] or mesenchymal transformation in endothelial cells [37]. Collectively, these changes result in cellular and molecular changes in the trabecular meshwork causing a reduction of outflow facility and hence raised IOP [38].

A reduction in the endothelial cell count has been demonstrated in different types of glaucoma. Three hypotheses have been formulated for this: damage from direct compression of the corneal endothelium because of higher IOP; alteration of both the corneal endothelial cell layer and the trabecular meshwork in patients with glaucoma (e.g., due to TGF-*β*); and glaucoma medication toxicity [39]. The relevance of endothelial cell loss in glaucoma is important to consider if patients are to undergo intraocular surgery.

Interestingly, in 1997, Gagnon et al. reported that despite the reduction in cell numbers, the morphology of corneal endothelial cells (including the percentage of hexagonal cells and coefficient of variation in cell area) did not differ significantly when different types of glaucoma patients were compared to controls [39]. Whilst increased intraocular pressure had been associated with deceased corneal endothelial cell density, no significant correlation between cell density and duration of the glaucoma has been identified [39, 40].  Table 1 provides a summary of corneal endothelial density changes in different forms of glaucoma.

### 2.1. Angle Closure Glaucoma

Angle closure glaucoma is caused by obstruction of the trabecular meshwork by iris tissue, which prevents the drainage of aqueous humour and therefore a rise in IOP in the eye, which often results in optic nerve damage [41]. Corneal endothelial cell loss has been frequently reported after acute angle closure glaucoma (AACG) [42–48] and chronic angle closure glaucoma (CACG) [46, 49]. Multivariate analysis for AACG found that duration of the acute attack was the only factor independently associated with reduced corneal ECD (*p* < 0.001) [47]. As demonstrated in a study which analysed AACG patients into two groups, an AACG attack was less than 72 hour durations or more than 72 hours duration [46]. Mean endothelial cell count in eyes which had a shorter duration (<72h) was 2016 ± 306 cells/mm^2^ compared to 759 ± 94 cells/mm^2^ in those who had AACG for more than 72 hours (*p* < 0.001) [46]. Two more recent studies which evaluated the cell count and morphological characteristics of corneal endothelial cells revealed no clinically significant differences across the angle closure disease spectrum (primary angle closure suspect, primary angle closure glaucoma, and previous acute angle closure glaucoma) [50, 51].

### 2.2. Open Angle Glaucoma

High tension primary open angle glaucoma (HTG) patients have a raised IOP despite an anatomically unoccluded angle, which results in optic nerve damage. In normal tension glaucoma (NTG), patients demonstrate optic nerve damage despite having a normal intraocular pressure and an open angle. Research demonstrates that there is a reduction in corneal endothelial cell density in HTG; however, the limited analyses of these changes when compared to NTG present conflicting findings [40, 48, 52]. One group found comparable cell counts between NTG and HTG patients: 2,343 ± 394 and 2,326 ± 231 cells/mm^2^, respectively [48]. Whilst others have reported significantly lower endothelial cell counts in NTG versus HTG patients (2,380.0 ± 315.4 vs. 2,530.0 ± 320.4 cells/mm^2^, *p* = 0.04), that is 6.3% less in NTG(54). Lee et al. postulated that in NTG a hypoperfusion mechanism accounted for both progressive optic neuropathy and endothelial cell density reduction [52].

Cho et al. found that the patients with HTG had a significantly lower endothelial cell density than controls (*p* < 0.001), but NTG patients had a similar cell density compared to controls [40]. The benefit of the Cho et al.'s study was that patients had no previous history of treatment with glaucoma medications. Analysis of 18,665 donor corneas received at the Lion's Eye Institute demonstrated that a past ocular history of glaucoma (in 2.7%) did not significantly affect endothelial cell density (*p* = 0.094), although the type of glaucoma was not specified [53].

### 2.3. Pseudoexfoliative Glaucoma

Pseudoexfoliative glaucoma (XFG) is the most common cause of open angle glaucoma worldwide [54, 55]. It is characterized by deposition of pathological greyish-white extracellular fibrillar protein components (PEX material) in multiple ocular tissues which is comprised of constituents of the basement membrane and elastic fibre components [56]. Deposition of this PEX material in the trabecular meshwork obstructs aqueous outflow and almost 50% of pseudoexfoliation syndrome (XFS) patients will ultimately develop XFG in their lifetime [57]. Electron microscopy has revealed large clumps of pseudoexfoliation material adhering to the corneal endothelium and this becomes incorporated into the posterior Descemet's membrane [58]; these may lead to early corneal endothelial decompensation. Patients with XFS and/or XFG have been consistently found in multiple studies to have lower corneal endothelial cell density than controls [59–68]. However, multiple groups have demonstrated that there is no significant difference between the endothelial cell density between patients with XFS alone compared to XFG [66].

Comparison of cell densities in all cell layers of the cornea have been found to be significantly lower in XFS eyes compared to age matched controls [63]. A Japanese study found a higher degree of pleomorphism and polymegathism in PEX eyes compared to control eyes, with the coefficient of variation of the cell area being significantly higher and the percentage of hexagonal cells was significantly lower in XFS [63]. Miyake et al. also demonstrated similar findings [69]; however, this was in contrast to another Japanese population [61] and in other regional studies in which there was no significant difference found in these coefficients of variation of cell size and frequency of hexagonality between XFS and control cataract patients: Paraguay population [65], Turkish population [67], and Chinese population [64]

## 3. Glaucoma Medications and Corneal Endothelium

Kwon et al. analysed the effect of topical medications used to treat glaucoma on the corneal endothelium in 134 donor corneas at the Lion's Eye Institute. No statistically significant reduction of ECD in patients on glaucoma medication was found. The mean ECD for donors not on glaucoma medication and pooled donors on glaucoma medication was 2561 ± 348 and 2516 ± 320 cells/mm^2^, respectively (*p* = 0.42) [76]. Analysis of ECD in patients on the ocular hypertensive treatment study (OHTS) demonstrated there was no statistically significant difference between those who had been observed for six years (*n* = 21) compared to those treated with any topical medications (*n* = 26) −2415 ± 300 compared to 2331 ± 239 cells/mm^2^, respectively (*p* = 0.6) [70]. There was no significant difference in the percentage of hexagonal cells between the two groups at six years either (*p* = 1.0). Other human studies have also not found a deleterious effect of topical glaucoma medications on ECD [77–79].

Gagnon et al. demonstrated that patients on three or four glaucoma medications had lower cell counts that patients receiving one or two medications [39]. This may be due to a correlation between disease severity and/or medication toxicity. Combined topical agents available for glaucoma treatment have also been analysed [73, 80, 81]. Two studies analysing the effects of latanoprost [80], brinzolamide/latanoprost [80, 81], and latanoprost/timolol [81] for shorter periods of two-three months also demonstrated no significant effect on corneal ECD.

Urban et al. analysed the difference in endothelial cell count in patients with juvenile open angle glaucoma treated with carbonic anhydrase inhibitor, prostaglandin analogue, beta blocker, and carbonic anhydrase inhibitor (CAI)/beta blocker combination. [73] They found no statistical difference in endothelial cell count between these four groups. Ayaki et al. exposed human cultured corneal endothelial cells to different glaucoma medications preserved and nonpreserved. They reported that cell viability in the presence of a commonly used preservative in eye drops (benzalkonium chloride) was markedly lower, especially with higher concentrations and longer exposure [82].

There has been concern over the use of carbonic anhydrase inhibitors and potential deleterious effects on the cornea. The corneal endothelium function relies on a bicarbonate pump to reduce corneal resurgence, for which carbonic anhydrase is a catalyst. However, central ECD cannot directly relate to endothelial function because of the significant functional reserve of this cell layer. No conclusive findings have been observed between carbonic anhydrase inhibitor use and corneal ECD loss [78, 79, 83].

Recently, there has been increasing interest in the use of Rho kinase inhibitors for glaucoma therapy due to the effects on the cytoskeleton of TM cells and Schlemm's canal cells which result in changes of cell morphology and permeability [84]. Netarsudil is the first Rho kinase inhibitor approved for glaucoma therapy in the US. Data from subjects who had 3 months of therapy with either netarsudil 0.02%/latanoprost 0.005% fixed combination (*n* = 126), netarsudil 0.02% (*n* = 143) only, or latanoprost 0.005% (*n* = 146) only compared to baseline found to have no significant difference or effect on ECD or morphology [85]. A significant decrease was observed in the central corneal thickness (CCT) in the fixed combination group (−6.4 *µ*m) compared to the two individual component groups (latanoprost (−1.2 *µ*m) or netarsudil (−3.3 *µ*m)), which may indicate that the potential effects of each drug on CCT are additive, although the magnitude of the observed effects is likely of negligible clinical significance [85].

A summary of changes observed in studies evaluating the effect of topical medications on corneal endothelial density is shown in Table 2. In conclusion, the active ingredients in topical ocular medications have little effect on the corneal endothelium [12]; however, the preservatives used within the medication can potentially affect corneal endothelial physiology [82].

## 4. Corneal Endothelium and Glaucoma Surgery

Endothelial cell damage and reducing ECD have been observed in most anterior segment procedures, including various types of glaucoma surgery [89]. Firstly, all implants within the anterior chamber can result in progressive endothelial cell loss [90] including glaucoma drainage devices, although the mechanism is unknown. Secondly, endothelial damage can be caused by a shallow or flat anterior chamber which occurs frequently after trabeculectomy or other filtering glaucoma surgeries [91]. Thirdly, the microinvasive glaucoma surgeries (MIGS) may cause damage related to their close proximity to the endothelium.

## 5. Glaucoma Drainage Devices (GDDs)

Numerous studies have evaluated endothelial cell loss after the implantation of tube drainage devices; however, varying methodologies used to quantify ECD, combination surgeries, and differing postoperative management strategies make it difficult to directly compare these studies.

### 5.1. Ahmed Valve

Statistically significant endothelial cell loss occurs following Ahmed valve implantation [90, 92–97]. Central corneal endothelial cell loss is reported to be between 7.6% and 11.5% (*p* < 0.05) at six months [90, 93, 94, 97], between 10.5% and 15.3% (*p* < 0.05) at 12 months [90, 93, 94] and one study reports 15.4% (*p* < 0.05) at 24 months [94]. A five-year retrospective case series reported that the cumulative risk of corneal decompensation following Ahmed valve insertion is 3.3% [92]. The same study demonstrated accelerated corneal endothelial cell density loss in eyes that had an Ahmed valve compared to fellow glaucomatous eyes which were medically managed (decrease of 7.0%/year and 0.1%/year, respectively; *p* < 0.001) [92]. However, the rate of loss decreased over time and was no longer statistically significant after two years compared to the controls [92].

Although the exact mechanism causing corneal endothelial cell loss after tube surgery is unknown, it is likely to be multifactorial. For example, changes in the circulation patterns of aqueous humour due to the glaucoma tube have been shown to adversely affect the endothelial cell viability [98–102]. In addition, the glaucoma drainage device itself may induce a breach in the blood-aqueous barrier, either by intermittent tube-uveal touch and/or chronic trauma from intermittent tube-corneal touch caused by heavily rubbing the eye or forcefully blinking, resulting in an increase of influx of oxidative, apoptotic, and inflammatory proteins, potentially causing corneal endothelial damage [98, 101, 103, 104].

A two-year prospective study of 41 eyes evaluated corneal ECD in various locations of the cornea before and after Ahmed valve insertion [94]. After 24 months, the greatest loss was seen in the supratemporal area (22.6%), closest to the site of the tube, whereas the central cornea showed the smallest decrease (15.4%) [94]. A one-year study of 30 eyes reported similar results [90]. Another study of 33 eyes with superotemporally placed Ahmed valves used the difference between supratemporal and inferonasal ECD as an estimate of the change in total ECD [95]. Distance from the tip of the tube to the cornea was significantly associated with fewer endothelial cells superotemporally compared with inferotemporally. Each millimetre that the tube was closer to the endothelial surface was associated with 353.1 fewer endothelial cells superotemporally (*p* = 0.02) [95]. No significant change in the cell morphology has been reported, except one study that documents an increase in the polymegathism and pleomorphism of corneal endothelial cells in the early postoperative period, but these returned to baseline after six months [90, 94, 105]. In addition, a comparison of sulcus sited Ahmed valve compared to anterior chamber sited valves demonstrated that the mean monthly central endothelial cell loss was significantly higher in tubes sited in the anterior chamber [106, 107]. There was also a significant increase in endothelial cell size in anterior chamber tubes compared to those placed in the sulcus [107]. Furthermore, increasing age of the patient and tube location in the anterior chamber were significantly associated with faster endothelial cell loss [106]. These findings support the theory that tubes closer to the cornea potentially result in increased endothelial cell loss.

When compared to trabeculectomy, Ahmed valves have demonstrated significantly higher endothelial cell loss [93, 96]. In a prospective study of 40 eyes that had Ahmed valves inserted compared with 28 eyes that underwent trabeculectomy, mean central corneal endothelial cell density decreased by 9.4% at 6 months and 12.3% at 12 months compared with baseline values (both, *p* < 0.001) in the Ahmed valve group [93]. Whist the decrease was less marked in the trabeculectomy group, there was a 1.9% loss at 6 months and 3.2% loss at 12 months (*p* = 0.027 and *p* = 0.015, respectively) [93]. In the Ahmed valve group, there was a significant decrease in the corneal ECD between baseline to 6 months and between 6 and 12 months (*p* < 0.001 and *p* = 0.005, respectively). However, in the trabeculectomy group, a significant decrease was observed only between baseline to 6 months (*p* = 0.027) [93]. This study demonstrated that the corneal endothelial cell loss was not only greater in the Ahmed valve group but also persisted for longer. Another study involving 18 patients reported similar findings that corneal endothelial cell loss was statistically significant and higher in the Ahmed group compared to the trabeculectomy group (*p* > 0.001) [96].

### 5.2. Molteno Implant

A cohort study directly comparing Ahmed valves in 29 eyes with Molteno implants in 28 eyes demonstrated no significant difference in central corneal endothelial cell loss (11.52% and 12.37%, respectively) after 24 months [108]. They also noted minor increases in central corneal endothelial cell area for both implants. These findings suggest that the type of implant may not matter, rather the presence of a silicone tube in the anterior chamber.

### 5.3. Baerveldt Glaucoma Drainage Device

Two prospective studies have evaluated the effect of the Baerveldt (BV) glaucoma drainage device on the corneal endothelium [109, 110]. The first study found that after 36 months, central and peripheral corneal ECD had decreased by 4.54% per year and 6.75% per year, respectively (*p* < 0.001) [109]. Moreover, corneal endothelial cell loss was related to the distance from the tube, with patients with a shorter tube-corneal (TC) distance experiencing an annual loss of 6.20% in the central cornea and 7.25% in the quadrant closest to the BV compared to those with longer TC distances who had an annual loss of 4.11% in the central cornea and 5.77% in the quadrant closest to the BV (*p* < 0.001) [109].

A second recent study of 64 eyes found that the mean percentage central ECD and peripheral ECD loses at five years were 36.8% and 50.1%, respectively [110]. Tube insertion in the vicinity of, or anterior, to Schwalbe's line as well as a shorter tube length were significantly associated with endothelial cell loss over time [110]. This suggests significant corneal endothelial cell loss with Baerveldt glaucoma drainage devices, particularly in the quadrant closest to the valve.

## 6. Trabeculectomy

Surgical trauma produced by trabeculectomy and the adjuvant use of mitomycin C (MMC) reduces ECD. Indeed MMC has been found in the aqueous humour after trabeculectomy [111], the presence of which could inhibit periodic repair of DNA as human corneal endothelium is primarily a nonreplicative tissue [112]. Additionally, short-term exposure of human corneal endothelial cells to MMC has shown the formation and interaction of free radicals that cause corneal swelling and disruption of intracellular endothelial organelles [113].

A number of studies showed that ECD loss after trabeculectomy with MMC was 1.9% to 18% [105, 114–121]. However, the results were derived from a relatively small number of cases with short postoperative follow-up periods (i.e., most were 12 months). A study with a longer follow-up of 24 months found the mean ECD decrease was 9.3%, but subgroup analysis demonstrated this was higher in XFG (18.2%) and uveitic glaucoma (20.6%) compared to 1.8% in POAG [122]. Two prospective randomised clinical studies on humans demonstrated endothelial cell damage at 3 and 12 months after MMC trabeculectomy [114, 115], but a subsequent study confirmed significant cell loss occurs during or immediately after MMC-augmented trabeculectomy [123]. Additionally, the active endothelial adaptations observed with no change in ECD between 3 and 12 months suggests that MMC has no prolonged toxic effect on the corneal endothelium. The grade of iridocorneal touch after an overdraining trabeculectomy is also correlated with an increased reduction in ECD [91].

Use of an anterior chamber maintainer or an injection of viscoelastic into the anterior chamber during trabeculectomy might provide more protection for the corneal endothelial cells [120, 124].

## 7. Deep Sclerectomy

There is presently only one published study evaluating the changes in ECD after deep sclerectomy (DS) and trabeculectomy [116]. The authors reported a significant reduction in cell loss between sclerectomy and trabeculectomy, 2.6% vs. 7% in central cornea, and 3.3% vs. 10.6% in upper cornea, respectively. They hypothesized the reason for this difference is because DS is less invasive than trabeculectomy as it does not penetrate the anterior chamber. When either DS or trabeculectomy was combined with cataract surgery, the difference was not statistically significant [116]. It is important to remark that this study compared DS with trabeculectomy without the use of antimetabolites.

## 8. Microinvasive Glaucoma Surgeries (MIGS)

In the last 10 years, microinvasive glaucoma surgeries (MIGS) have been increasingly used as an approach for treating glaucoma. MIGS can be divided into three main groups: Schlemm's canal MIGS, suprachoroidal MIGS, and subconjunctival MIGS.

### 8.1. Schlemm's Canal MIGS

The iStent (Glaukos Corp., San Clemente, CA, USA) has shown a moderate effect in controlling IOP [125, 126]. In a series of 10 Japanese eyes with OAG undergoing standalone implantation of 2 first-generation iStents, no change in ECD was observed through 6 months of follow-up [127]. An evolution of the iStent, the iStent Inject, has been developed to increase the efficacy of this device [128]. The iStent Inject's pivotal trial evaluated ECD and found a 13.1% reduction at 24 months postoperatively in the iStent-phaco group compared to a 12.3% reduction in eyes going phacoemulsification only [128]. The majority of the reduction in the ECD occurred within the first 3 months [128]. Similarly, a further study found a reduction of 9.0% (*n* = 21) at a mean follow-up of 18.2 months, as well as a significant reduction in the percentage of hexagonal cells [128].

In a prospective, uncontrolled case series of 20 eyes undergoing combined iStent-phaco, mean ECD decreased from 2290 to 1987 cells/mm^2^ (13.2% decrease) at 12 months [129]. Evaluation of 12-month data after the implantation of 2 iStent Inject devices combined with phacoemulsification (*n* = 54) found a 14.6% reduction in the endothelial cell count from baseline (2417 ± 417 cells/mm^2^ at baseline to 2065 ± 536 cells/mm^2^ at 12 months, *p* = 0.001) which was comparable to patients undergoing phaco alone (-14.4%) [130].

Ivantis, Inc., (Irvine, CA, USA) developed a new device in 2014 called the Hydrus Microstent [131]. A retrospective nonrandomised clinical study comparing the endothelial changes after a Hydrus (Hydrus, Ivantis, Irvine, CA) MIGS implant combined with cataract surgery (*n* = 37) versus cataract surgery alone (*n* = 25) did not show any difference in endothelial parameters 6 months [132]. The HORIZON study found that the ECD reduced from 2417 ± 390 cells/mm^2^ at baseline to 2056 ± 483 cells/mm^2^ at 3 years in the combined phacoemulsification and Hydrus (*n* = 369) group compared to a reduction from 2426 ± 371 cells/mm^2^ at baseline to 2167 ± 440 cells/mm^2^ at 3 years in the phaco alone group (*n* = 187) [133]. This reduction was initially related to the surgical procedure and the addition of the Microstent induced an incremental nonsignificant loss in mean central cell count of 2% (approximately 75 cells/mm^2^) [133]. This finding may be related to the additional surgical manipulation with insertion and removal of additional cohesive viscoelastic when placing the device. Sequential visit-to-visit changes in endothelial cell counts were consistent between the study groups and this was not statistically significant [133]. After the initial loss in cell count related to the surgery, no difference was found in the year-to-year change in the proportion of eyes with 30% endothelial cell loss between groups [133].

### 8.2. Suprachoroidal MIGS

Suprachoroidal MIGS target the uveoscleral pathway to reduce the IOP. Cypass (Alcon, Ft. Worth, TX, USA) [134], a suprachoroidal MIGS was unfortunately recalled in 2018 as the 5-year data demonstrated high rates of endothelial cell loss (3% per year in the Cypass group compared to 1% control phaco alone) that were deemed to compromise its safety [135]. At month 60, the mean percent of changes in ECD was −20.4% (95% CI, −23.5% to −17.5%) in the phaco and Cypass group (*n* = 282) and −10.1% (95% CI, −13.9% to −6.3%) in the control group (*n* = 67) [135]. In addition, 9 adverse events were possibly related to ECD loss, including 3 eyes with transient focal corneal oedema and 4 eyes that required Cypass trimming due to protrusion. The prominent position of the device within the anterior chamber was deemed to be the reason for the changes observed and in some instances the Cypass stent has been explanted due to corneal decompensation [136].

### 8.3. Subconjunctival MIGS

Subconjunctival MIGS include the XEN subconjunctival implant gel stent (Aquesys, Aliso Viejo, CA, USA/Allergan, Irvine, CA, USA). One study evaluated standalone phacoemulsification (*n* = 15) and found a mean reduction of ECD by 14.5% at 24 months compared to a mean reduction of 14.3% at 24 months in the combined phaco/XEN surgery (*n* = 17). The difference in percentage reduction of ECD between the 2 groups was not significant (*p* = 0.226) [137]. A further study compared trabeculectomy (*n* = 31) to XEN gel stents (*n* = 49) and found a significantly higher rate of cell loss at 3 months in the trabeculectomy group (-10%) than the XEN gel stent group (-2.1%) when compared to baseline [138].

In recent years, the Preserflo (formerly InnFocus) (Santen Co., Japan), which creates a bleb by the insertion of an 8.5 mm polymeric in anterior chamber via a scleral pocket, has come to the market. Results showed no significant difference at 6 months between endothelial cell loss in 26 eyes with Preserflo (gain of 2.7%) compared to 26 after trabeculectomy (loss of 3.2%) [139]. Both procedures significantly changed the coefficient of variation but had no significant changes on percentage of hexagonal cells. The endothelial cell count was evaluated at one year as part of a 2 year prospective randomised multicentre study of the Microshunt (*n* = 395) versus trabeculectomy (*n* = 132) [140]. Endothelial cell loss was similar in both groups at year 1 (05.2% after Microshunt implantation and -6.9% after trabeculectomy). One patient in the Microshunt group experienced endothelial cell loss of 9.4% between 6 months and 1 year, which was presumed to be due to the proximity of the device to the cornea [140].

The Ex-Press mini glaucoma shunt (Alcon Laboratories, Fort Worth, TX) is a further subconjunctival Microshunt. Studies have compared the Ex-Press shunt with trabeculectomy and Ahmed valves [105, 119]. In a 3-month prospective study, no significant reduction in corneal ECD occurred in the Ex-Press group (1.3%, *p* > 0.05) [105]. Unlike the trabeculectomy group which had a significant decrease of 3.5% at 1 month (*p* = 0.012) and 4.2% at 3 months (*p* = 0.007), and the Ahmed valve group, where a significant decrease of 3.5% was seen after 3 months (*p* = 0.04) [105], a further group found reduction of endothelial cell count after Ex-Press implantation by 3.5%, but no significant difference between trabeculectomy and the Ex-Press shunt [119]. Other groups, however, have demonstrated cases of corneal decompensation after the Ex-Press stent and significant reductions of endothelial cell count (4% at 24 months from baseline), which may have been due to intermittent endothelial contact [141, 142]. In addition, the endothelial cell loss has been observed to be significantly higher in the superior cornea, which is close to the shunt site (-17.6%) compared to the inferior cornea (-11.7%) [143].

Alternative MIGS interventions include Ab interno-trabeculotomy with the Trabectome device (NeoMedix, Tustin, CA, USA) which has been shown to have minimal effects on corneal endothelial cells at 6 months and up to 36 months postoperatively [144, 145]. A goniotomy with the Kahook Dual Blade (KDB, New World Medical, Rancho Cucamonga, CA) has been shown to reduce the endothelial cell density by only 3.4% at a mean follow-up of 18.2 months after procedure (*n* = 21) with no significant effect on other morphological parameters [146]. Furthermore, the Excimer Laser Trabeculotomy (ELT, Glautec AG, Nurnberg, Germany) [147], the Fugo Blade (MediSurg Research and Management Corp., Norristown, PA, USA) [98, 148], the Ab interno-canaloplasty (ABIC) [99, 100], and the gonioscopy-assisted transluminal trabeculotomy (GATT) [101] could potentially have an impact on the endothelial cell count. No studies are presently available in the literature in regard to these.

## 9. Descemet Membrane Endothelial Keratoplasty (DMEK) Use in the Management of Glaucoma-Related Endothelial Cell Loss

Corneal endothelial cell loss can subsequently result in corneal decompensation, and this continues to be a common comorbidity after glaucoma surgery [102]. The introduction of Descemet stripping automated endothelial keratoplasty (DSAEK) and Descemet membrane endothelial keratoplasty (DMEK) has replaced the use of penetrating keratoplasty (PK) as the standard of care for endothelial disorders [103]. In the presence of glaucoma drainage devices, higher rates of corneal graft failure and increased ECD loss are observed after penetrating keratoplasty and DSAEK; as suggested earlier, the reasons for this are multifactorial [104, 149–153].

DMEK surgery is increasingly used as a method of treating corneal endothelial dysfunction and shows reduced rejection rates and faster visual recovery when compared to DSAEK [154–156]. A key benefit is that the rapid visual recovery and reduction in corneal oedema allows for early visual field testing or optic nerve examination to decide on further glaucoma management [157]. Another advantage of DMEK is that the taper of topical corticosteroids postoperatively is quicker than that after PK and DSEK. The quicker taper potentially lowers the risk of IOP elevation, resulting from the steroid response [158]. The steroid IOP response rates after DMEK and DSAEK have been shown to be 15% and 17%, respectively (*p* = 0.768) [159, 160]. These are not any higher than expected for any patient on long-term steroidal treatment [159, 160].

Performing a DMEK surgical procedure is, however, more challenging in eyes with previous glaucoma surgery. For example, the presence of corneal oedema, a tube shunt, anterior synechiae, previous trabeculectomy, or an abnormal anterior segment can make the surgery more difficult [157]. Studies have been performed to evaluate the outcomes and complications of DMEK surgery after glaucoma surgery, as summarized in Table 3.

## 10. Conclusions

In summary, we have outlined the endothelial cell changes which occur due to glaucoma itself, as well as those which occur as a result of its medical and surgical management, including new generation MIGS devices. We have explored the use of DMEK for the management of corneal endothelial failure and the recent literature illustrating the results including complications after performing DMEK for postglaucoma endothelial loss. Additional studies are required to investigate the cause of the accelerated endothelial cell loss in glaucoma patients undergoing DMEK surgery and assessment of glaucoma progression related to DMEK surgery.

## Figures and Tables

**Table 1:** Corneal endothelial densities in different forms of glaucoma.

	Control mean (cells/mm^2^)	Control SD (cells/mm^2^)	No. controls	Cases mean (cells/mm^2^)	Cases SD (cells/mm^2^)	No. of cases	*P* value
*Ocular Hypertension*							
Baratz et al., 2006 [70]	2415	300	21	2331	239	26	0.6
Chawla et al., 2021 [71]	2509.1	298.5	91	2559.8	268.2	8	0.588

*All forms of glaucoma*							
Gagnon et al., 1997 [39]	2560	306	52	2154	419	102	<0.0001
Novak Stroligo et al., 2010 [68]	2528	306	100	2148	317	100	<0.0001

*Acute PACG*							
Setala et al., 1979 [43]	2392	346	25	2161	633	25	N/A
Bigar et al., 1982 [44]	2243	N/A	20	1534	N/A	20	0.002
Malaise-Stals et al., 1984 [45]	2398	380	174	1640	N/A	44	N/A
Chen et al., 2012 [47]	2559	50	50	2271	80	40	0.002
Sihota et al., 2003 [46]	2461	321	30	1597	653	30	<0.001
Verma et al., 2018 [50]	N/A	N/A	N/A	2504.0	558.1	74	N/A

*Subacute ACG*							
Sihota et al., 2003 [46]	2461	321	30	2396	271	30	<0.001

*PACG-unspecified*							
Gagnon et al., 1997 [39]	2560	306	52	2000	585	30	<0.0001

*PACS*							
Varadaraj et al., 2017 [51]	N/A	N/A	N/A	2676.8	270.0	466	N/A
Verma et al., 2018 [50]	N/A	N/A	N/A	2582.0	472.8	51	N/A

*CACG*							
Tham et al., 2006 [49]	N/A	N/A	N/A	2271.7	312.9	39	N/A
Chen et al., 2012 [47]	2559	50	50	2379	50	44	0.316
Sihota et al., 2003 [46]	2461	321	30	2229	655	30	<0.001
Varadaraj et al., 2017 [51]	N/A	N/A	N/A	2681.2	275.7	127	N/A
Verma et al., 2018 [50]	N/A	N/A	N/A	2523.8	406.8	234	N/A
Chawla et al., 2021 [71]	2509.1	298.5	91	2378.2	677.9	13	0.588

*ACG Unspecified*							
Novak Stroligo et al., 2010 [68]	2528	306	100	2113	243	24	N/A

*NTG*							
Lee et al., 2015 [52]	N/A	N/A	N/A	2380	315.4	30	N/A
Cho et al., 2009 [40]	2723.6	300.6	91	2696.7	303.9	87	1
Chawla et al., 2021 [71]	2509.1	298.5	91	2420.6	515.7	19	0.588

*HTG*							
Gagnon et al., 1997 [39]	2560	306	52	2226	311	55	<0.0001
Cho et al., 2009 [40]	2723.6	300.6	91	2370.5	392.3	49	<0.001
Lee et al., 2015 [52]	N/A	N/A	N/A	2530	320.4	28	N/A
Yu et al., 2019 [72]	2959	236	60	2757	262	60	<0.001
Chawla et al., 2021 [71]	2509.1	298.5	91	2517.9	245.3	39	0.588

*ACG Unspecified*							
Knorr et al., 1991 [59]	2302	394	4432	1812	297	123	<0.001
Seitz et al., 1995 [60]	2372	276	33	2214	251	16	N/A
Inoue et al., 2003 [61]	2362	327	30	2337	407	19	N/A
Wali et al., 2009 [62]	2460	N/A	N/A	2483	511.2	78	N/A
Zheng et al., 2011 [63]	2738.7	233.3	27	2240.7	236.6	27	<0.0001
Wang et al., 2012 [64]	2562	18	20	2505	284	7	N/A

*XFS and senile cataract*							
Quiroga et al., 2010 [65]	2482	N/A	356	2315	N/A	61	0.002
Tomaszewski et al., 2014 [66]	2503	262	84	2297	359	68	0.0008
Bozkurt et al., 2015 [67]	2363	229.3	51	2299.5	213.9	33	0.48
*PXG and senile cataract*							
Tomaszewski et al., 2014 [66]	2503	262	84	2241	363	65	0.000005
Bozkurt et al., 2015 [67]	2363	229.3	51	2199.5	176.8	19	0.02

*PXG*							
Knorr et al., 1991 [59]	2302	394	4432	1482	267	59	<0.001
Seitz et al., 1995 [60]	2372	276	33	2014	254	69	N/A
Inoue et al., 2003 [61]	2362	327	30	2332	336	7	N/A
Wali et al., 2009 [62]	2460	N/A	N/A	2438	503.4	48	N/A
Novak Stroligo et al., 2010 [68]	2528	306	100	2024	254	16	<0.0001
Wang et al., 2012 [64]	2562	18	20	2186	2	13	N/A
Chawla et al., 2021 [71]	2509.1	298.5	91	2392.2	258.4	12	0.588

*Juvenile Open Angle Glaucoma*							
Urban et al., 2015 [73]	2955.5	N/A	33	2639.5	N/A	66	<0.0001

*Congenital glaucoma*							
Guigou et al., 2008 [74]	3470	357	401	2922	553	69	<0.001

*Congenital and secondary juvenile glaucoma*							
Wenzel et al., 1989 [75]	N/A	N/A	N/A	2780	N/A	20	N/A

SD, standard deviation; PACG, primary angle closure glaucoma; PACS, primary angle closure suspect; CACG, chronic angle closure glaucoma; NTG, normal tension glaucoma; XFS, pseudoexfoliation syndrome; PXG, pseudoexfoliation glaucoma; HTG, high tension primary open angle glaucoma.

**Table 2:** Effect of topical medication on corneal endothelial cell density (CECD).

	% mean cell CECD change at 1 year to baseline (SD)	Number of patients	Citation
Prostaglandin analogues			
Latanoprost	0.3 (2.2)	127	[86]
	−2.3	18	[87]
	−3.2 (6 months)	54	[88]
	−0.04 (3 months)	146	[85]

Carbonic anhydrase inhibitor			
Dorzolamide	No significant difference		[79]
	0.2	7	[78]
	−3.6 (5.0)	148	[83]

Beta blocker			
Timolol	−4.5 (4.2)	72	[83]
	0.1 (1.8)	126	[86]
Betoxalol	−4.2 (3.6)	78	[83]

Rho Kinase Inhibitor			
Netarsudil 0.02%	0.6 (3 months)	143	[85]

Combined therapy			
Latanoprost-timolol	0 (2.5)	126	[86]
Latanoprost-brinzolamide	−0.6	16	[87]
Netarsudil 0.02%/latanoprost	0.6 (3 months)	126	[85]

**Table 3:** A table to show the previous studies performed evaluating the clinical outcomes of DMEK after glaucoma surgery.

Paper	Number of patients	Previous glaucoma surgery	Length of follow-up	VA improvement	Rate of ECD loss postop	Primary graft failure	Rebubbling rates	Secondary graft failure	Postoperative IOP
Alshaker et al., 2021 [160]	*N* = 48 DMEK, *N* = 41 DSAEK	62.5% tube, 37.5% trab only, 61.0% tube, 39.0% trab only	30.0 ± 15.5 months, 33.9 ± 22.5 months	Postoperative BCVA was significantly better in DMEK group at 24 months (*p* = 0.047)	57%, 50% (*p* = 0.886) at 36 months	14.6%, 14.6% (*p* = 1.0)	25.0%, 19.5% (*P* = 0.615	20.8%, 19.5% (*p* = 1.0)	14.6%, 17.1% (IOP: 25–45 mmHg) (*P* = 0.768)
Fili et al., 2021 [161]	*N* = 16 DMEK, *N* = 9 DSAEK, *N* = 15 PK	68.8% trab, 43.8% tube, 100.0% trab, 22.2% tube, 80.0% trab, 40% tube,	24 months	Final BCVA at 24 months 0.34 ± 0.29 0.17 ± 0.18 0.06 ± 0.07	50.7%, 48.9%, 42.7% at 12 months (*P* < 0.45)	6.25%, 11.11%, 0	N/A	12.5%, 33.33%, 13.33%	6.25%, 11.11%, 0
Bonnet et al., 2020 [158]	*N* = 11 medically treated GL, *N* = 38 surgically treated Gl, *N* = 41 no GL	55.3% previous trab, 68.4% previous tube	38.4 ± 11.2 months	Achieved BCVA of ≥20/20, 46%, 8%, 49% **(p** **<** **0.01)**	47.6%, 63.8%, 44.0% **(p** **<** **0.05)**	0%, 0%, 0%	18.2%, 15.8%, 19.5% (*p* = 0.93)	0%, 41.6%, 2.4% **(p** **<** **0.05)**	54.5%, 26.3%, 34.1% (IOP >24 mm Hg or >8 increase) (*p* = 0.21)
Sorkin et al., 2020 [162]	*N* = 51 surgically treated Gl, *N* = 43 controls	49.0% previous tube, 13.7% trab and tube, 37.3% previous trab	37.9 ± 15.2 months, 33.8 ± 13.5 months	Mean BCVA improved from 1.82 ± 0.88 logMAR preop to 1.06 ± 0.87 at 6 months (no comparison to controls)	74%, 52% at 48 months **(P** **=** **0.004)**	15.7%, 11.6% *P* = 0.766	23.5%, 30.2% *P* = 0.491	47.1%, 0% **P** **<** **0.001**	7.8% (control group not reported)
Boutin et al., 2020 [163]	*N* = 27	44.4% prior tube, 33.3% prior trab, 14.8% trab and tube, 3.7% trab and gold Microshunt, 3.7% prior tube and hydrus	14.6 ± 6.1 months	Mean BCVA improved from 1.34 ± 0.65 logMAR preop to 0.50 ± 0.33 at 1 year	50.4% at one year **(P** **=** **0.001)**	3.7%	18.5%	10.3%	3.7%
Lin et al., 2019 [164]	*N* = 46 DMEK *N* = 46 DSEK	48% prior trab, 78% prior tube, 50% prior trab, 74% prior tube	12 months	Improved by -0.89 logMAR, -0.62 logMAR **(p** **=** **0.005)**	N/A N/A	2% 2% (*p* = 0.65)	22% 9% (*p* = 0.14)	0% 17% **(p** **=** **0.006)**	30% IOP elevation, 36% IOP elevation (increase >8 mmHg) (*p* = 0.66)
Birbal et al., 2018 [153]	*N* = 23	65% trabeculectomy, 100% tubes (85% 1 tube, 15% 2 tubes)	19 ± 17 months	BCVA improved by ≥ 2 Snellen lines in 73%	71%	8.7%	21.7%	8.7%	9% (IOP >24 mmHg or >10 mmHg increase)
Aravena et al., 2017 [165]	*N* = 14 medically treated GL, *N* = 34 surgically treated Gl, *N* = 60 no GL	52.9% tube only, 14.7% trab and tube, 32.3% trab only	9.7 ± 7.3 months	Achieved BCVA of ≥20/25, 71.4%, 32.4%, 62.6% **P** **<** **0.0001**	29.9 ± 12.0%, 44.6 ± 17.8%, 32.7 ± 11.3%, **P** **=** **0.001**	0, 0, 1.7% *P* = 1.0	21.4%, 23.5%, 23.3%	0, 0, 0	50.0%, 14.7%, 23.3% **(p** **=** **0.001)**

*N*: number; DMEK: Descemet membrane endothelial keratoplasty; DSAEK: Descemet striping automated endothelial keratoplasty; BCVA: best corrected visual acuity; ECD: endothelial cell density; IOP: intraocular pressure; GL: glaucoma.
